# Changes in the AGE/Macrophage/TNF-α Pathway Affect Skin Dryness during KK-Ay/Tajcl Mice Aging

**DOI:** 10.3390/life13061339

**Published:** 2023-06-07

**Authors:** Keiichi Hiramoto, Masashi Imai, Shota Tanaka, Kazuya Ooi

**Affiliations:** Department of Pharmaceutical Sciences, Suzuka University of Medical Science, Suzuka 513-8670, Japan; hiramoto@suzuka-u.ac.jp (K.H.);

**Keywords:** advanced glycation end products, collagen, macrophage, receptors of AGE tumor necrosis factor-α, type 2 diabetes

## Abstract

Skin dryness associated with type 2 diabetes worsens with age; however, the underlying mechanisms remain unclear. Herein, we investigated the effects of aging on skin dryness using a type 2 diabetes mice model. Specific pathogen-free KK-Ay/TaJcl mice of different ages (10, 27, 40, and 50 weeks) were used in this study. The results confirmed that skin dryness worsens with age. Furthermore, increased levels of advanced glycation end products (AGE), prostaglandin E2 (PGE2), and tumor necrosis factor (TNF)-α, along with an increased expression of the major AGE receptor (RAGE), an increased macrophage number, and decreased collagen expression were observed in the skin of aged KK-Ay/TaJcl mice. In conclusion, dry skin conditions worsen with age in diabetic mice, and the AGE/RAGE/PGE2 and TNF-α pathways play an important role in exacerbating skin dryness during aging in these mice.

## 1. Introduction

Diabetes, a chronic systemic disease, can lead to serious complications due to persistent hyperglycemia, thus lowering the quality of life of patients and shortening their life span [1]. Diabetes can worsen skin conditions and is associated with recurrent skin infections [2]. Neuropathy, a complication of type 2 diabetes, aggravates skin symptoms in patients with diabetes. Neuropathy is associated with reduced sweating, which ultimately causes skin dryness, cracking, and impaired barrier function [3]. Furthermore, diabetes-induced obesity promotes the migration of macrophages into the adipose tissue [4]. These macrophages secrete inflammatory cytokines, which, in turn, damage the skin [5].

Sustained hyperglycemia induces an irreversible glycation reaction (Maillard reaction) in which the excess sugar binds to proteins present in the blood vessels [6] leading to the accumulation of glycated proteins. Native and degraded glycated proteins are together known as advanced glycation end products (AGEs), which cause various adverse effects on the body [7]. AGEs bind to the major AGE receptor (RAGE) and activate the intracellular signaling pathways that mediate inflammatory responses [8]. AGEs can stimulate the retinal vascular endothelial cells to produce vascular endothelial growth factor (VEGF) and promote angiogenesis, thereby inducing diabetic proliferative retinopathy [9]. Moreover, AGE accumulation has also been observed in the kidneys of patients with diabetic nephropathy [10]. AGE accumulation in the skin collagen of diabetic patients is higher than that of healthy individuals, and this accumulation increases with age. The skin elasticity of diabetic patients is lower than that of healthy subjects [11], and this tendency is more pronounced in aged patients [12]. Thus, AGE accumulation in diabetic individuals greatly affects skin aging; however, the underlying mechanism remains unclear.

Herein, we used a type 2 diabetes KK-Ay/TaJcl mice model to investigate the role of AGE-mediated molecular changes in skin dryness exacerbation during aging and the relationship between aging, diabetes, and skin dryness.

## 2. Materials and Methods

### 2.1. Experimental Animals

Specific-pathogen-free (SPF) C57BL/6j and KK-Ay/TaJcl male mice (C57BL/6N background) of different ages (10, 27, 40, and 50 weeks) were obtained from CLEA Japan Inc. (Tokyo, Japan). C57BL/6j mice were used as control mice. Mice were caged individually in an air-conditioned room at 23 ± 1 °C under SPF conditions with a 12 h light/12 h dark cycle. Food and water were provided ad libitum. Body weight (g) of each mouse was measured every week. Blood samples from the tail vein were collected to measure the blood glucose levels. Animals with a blood sugar level of more than 300 mg/dL were considered diabetic. This study was approved by the Suzuka University of Medical Science Animal Experiment Ethics Committee on 25 September 2014 and performed in strict accordance with the recommendations of the Guide for the Care and Use of Laboratory Animals of the Suzuka University of Medical Science (approval number: 34). All surgeries were performed under pentobarbital anesthesia, and efforts were made to minimize animal suffering.

### 2.2. Measurement of Transepidermal Water Loss (TEWL) and Capacitance of the Dorsal Skin

TEWL and skin hydration were evaluated using the dorsal skin of all animals four weeks after initiating the experiment according to a previously described method [13,14]. Hair was shaved 24 h before measuring TEWL and skin hydration. In addition, it was confirmed that the mice skin returned to its original state 2 h after shaving. The presence or absence of inflammation was observed. TEWL is a measure of permeability that reflects the barrier function of the skin. Tewameter TM300 (Courage + Khazaka Electronic GmbH, Cologne, Germany) and Corneometer CM825 (Courage + Khazaka Electronic GmbH) were used to evaluate TEWL and skin hydration levels, respectively. The degree of hydration was evaluated by measuring the electrical capacitance of the skin and represented in terms of arbitrary units. For all measurements, the same pressure and place were used for each mouse.

### 2.3. Preparation and Staining of the Dorsal Skin

For histological analysis, mice were sacrificed, and dorsal skin was collected. The skin was fixed in 4% phosphate-buffered paraformaldehyde and embedded in Tissue-Tek OCT compound (Sakura Finetek, Co., Ltd. Tokyo, Japan). After, 5 μm thick sections were cut and air-dried at room temperature. The sections were stained with hematoxylin-eosin (HE) using the standard protocol. To analyze collagen expression, sections were stained using the Trichrome Stain Kit (Modified Masson’s) (ScyTec Laboratories, Inc., Logan, UT, USA) according to the manufacturer’s protocol. [15]. For immunofluorescence analysis, skin sections were stained with antibodies as previously described [16]. The following primary antibodies (1:100 dilution) were used: mouse monoclonal anti-AGEs (Medicinal Chemistry Pharmaceutical Co., Ltd., Kobe, Hyogo, Japan), rabbit polyclonal anti-RAGE (Abcam, Cambridge, MA, USA), rat monoclonal anti-F4/80 (marker of macrophage; Novus Biologicals, LLC., Centennial, CO, USA), rabbit polyclonal anti-monocyte chemoattractant protein (MCP)-1 (macrophage migration factor; Bioss Inc., Woburn, MA, USA), and anti-goat polyclonal anti-CC chemokine receptor 2 (CCR2) (receptor of MCP-2; GeneTex Inc., Irvine, CA, USA). The sections were then incubated with the appropriate secondary antibodies (1:30 dilution; fluorescein isothiocyanate-conjugated anti-rabbit, anti-mouse, anti-rat, or anti-goat secondary antibodies (Dako Cytomation, Glostrup, Denmark)) for 2 h in the dark. Collagen type I, AGEs, RAGE, F4/80, MCP-1, and CCR2 were calculated from five random visual fields with constant area using ImageJ software ver. 1.53 (National Institutes of Health, Bethesda, MD, USA). Briefly, original files were converted to monochrome 8-bit file. Next, the threshold of luminous intensity was voluntarily established. Areas above the threshold were measured in each sample. These areas were defined as “intensity” in this study.

### 2.4. Analysis of Skin Prostaglandin E2 (PGE2), Tumor Necrosis Factor (TNF)-α, Interleukin (IL)-1β, and Cyclooxygenase (COX)-2 Levels via Enzyme-Linked Immunosorbent Assay (ELISA)

Dorsal skin was isolated and homogenized in the lysis buffer (Kurabo, Osaka, Japan). The tissue extract was centrifuged at 10,000 rpm using a Tomy MX-201. The expressions of prostaglandin E2 (PGE2) and tumor necrosis factor (TNF)-α were measured using PEG2 (Abcam) and TNF-α (Enzo Life Sciences, Farmingdale, NY, USA) ELISA kits, respectively, following the manufacturers’ instructions. Optical density was measured using a microplate reader (Molecular Devices, Sunnyvale, CA, USA).

### 2.5. Statistical Analysis

All data are expressed as the mean ± standard deviation. Statistical analysis was performed using Microsoft Excel 2010. SPSS version 20 (SPSS Inc., Chicago, IL, USA) was used for one-way analysis of variance (ANOVA) and Tukey’s post hoc test. A *p*-value < 0.05 was considered statistically significant.

## 3. Results

### 3.1. Effect of Aging on Body Weight and Blood Glucose Level of KK-Ay/Tajcl Mice

**Figure 1:**
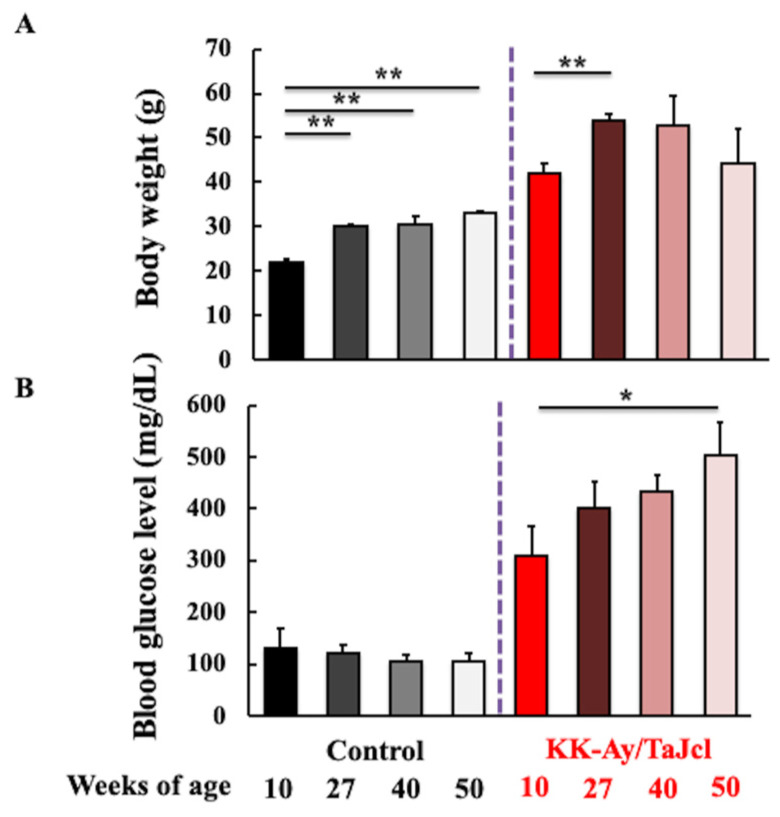
Effect of aging on body weight (**A**) and blood glucose levels (**B**) in control and KK-Ay/TaJcl mice. Values are expressed as the mean ± SD of five animals. * *p* < 0.05; ** *p* < 0.01. Control: C57BL/6j mice.

To confirm the effect of age on diabetes in KK-Ay/TaJcl mice, we examined body weight and blood glucose levels. Figure 1 shows the body weight (Figure 1A) and blood glucose levels (Figure 1B) of control and diabetic mice at different ages. In the control group, the body weight increased until 27 weeks of age and remained unchanged thereafter. In KK-Ay/TaJcl mice, the body weight peaked at 27 weeks of age and then decreased. Blood glucose levels did not change with age in the control group, whereas in KK-Ay/TaJcl mice, blood glucose levels were already high at 10 weeks of age and increased further with aging. These data suggest that aging exacerbates diabetes. In addition, KK-Ay/TaJcl mice were clearly diabetic as their blood glucose levels were higher than 300 mg/dL.

### 3.2. Effect of Aging on TEWL, Skin Hydration and Thickness, and the Expression of Collagen in KK-Ay/TaJcl Mice

**Figure 2:**
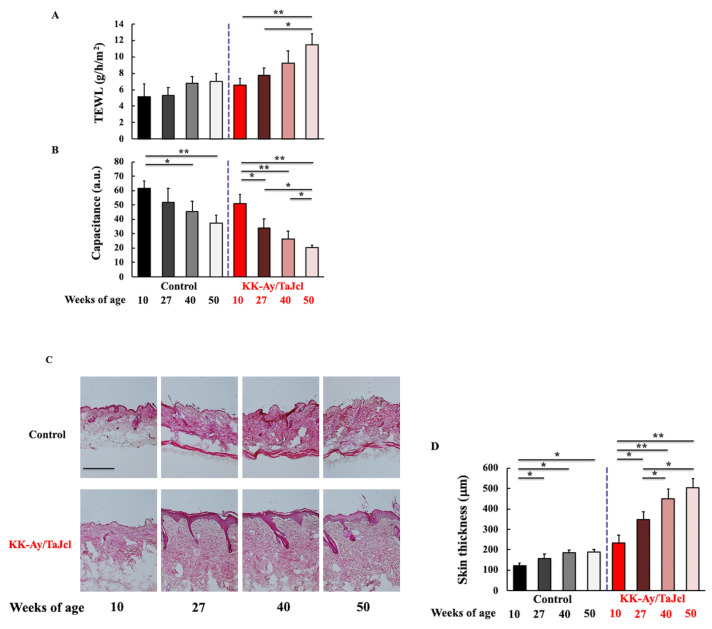

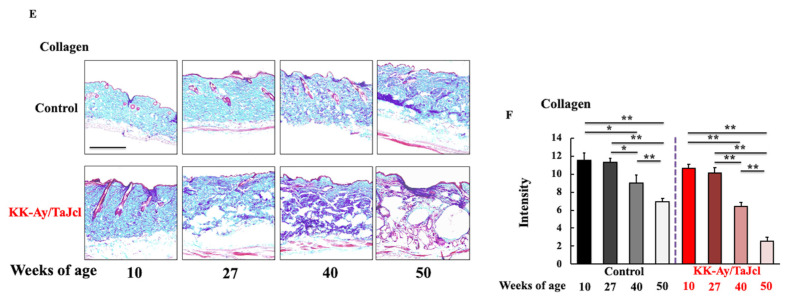
Effect of aging on transepidermal water loss (**A**), skin hydration (**B**), skin thickness (**C**,**D**), and skin expression of collagen (**E**,**F**) in KK-Ay/TaJcl mice. Hematoxylin-eosin staining (**C**) and Masson trichrome staining of collagen (**E**). Intensity was calculated from five random visual fields with constant area using the ImageJ software. TEWL, transepidermal water loss; a.u., arbitrary units. Values are expressed as the mean ± SD of five animals. Scale bar = 100 μm. N = 5. ** *p* < 0.01; * *p* < 0.05. Control: C57BL/6j mice.

To evaluate the effect of aging on skin dryness in diabetes mice, we analyzed TEWL (Figure 2A) and skin hydration levels (Figure 2B). Higher TEWL values correspond to drier skin. The control group showed no change in skin dryness with age, whereas in KK-Ay/TaJcl mice, skin dryness increased with age. Skin hydration decreased with age in both the control and KK-Ay/TaJcl groups, indicating dryness. Furthermore, in the control group, skin thickness increased until 27 weeks of age and did not change thereafter. Conversely, in KK-Ay/TaJcl mice skin thickness kept increasing with age (Figure 2C,D). In addition, the dermal expression of collagen decreased after 40 weeks of age in both control and KK-Ay/TaJcl mice groups. However, the decrease in KK-Ay/TaJcl mice was more pronounced (Figure 2E,F). These results suggest that skin dryness was exacerbated by aging in diabetic mice.

### 3.3. Effect of Aging on Skin Levels of AGEs, F4/80, and RAGE in KK-Ay/TaJcl Mice

**Figure 3:**
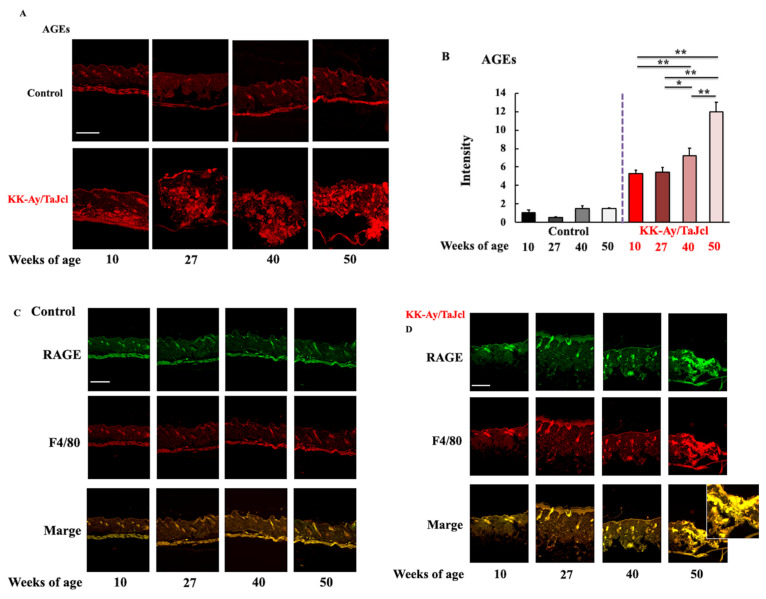

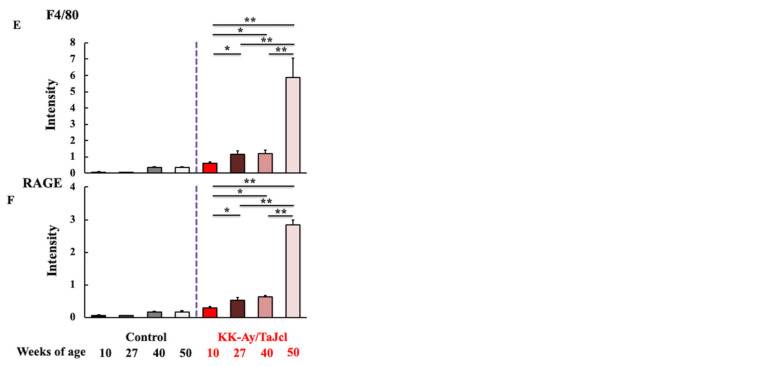
Effect of aging on AGE levels (**A**,**B**), RAGE expression (**C**,**D**,**F**), F4/80 (macrophage marker) expression (**C**–**E**), and the colocalization of RAGE and macrophage marker (**C**,**D**) in the dorsal skin of KK-Ay/TaJcl mice. Scale bar = 100 μm. Intensity was calculated from five random visual fields with constant area using ImageJ software. Values are expressed as the mean ± SD of five animals. N = 5. ** *p* < 0.01; * *p* < 0.05. Control: C57BL/6j mice.

To investigate the effects of AGEs on skin dryness in aging diabetic mice, we analyzed the levels of AGEs and RAGE and evaluated the number of macrophages in the skin. F4/80 was used as macrophage marker. Notably, AGE and RAGE levels and the number of macrophages were higher in the KK-Ay/TaJcl mice compared to in the control mice. Furthermore, AGE and RAGE levels and the number of macrophages markedly increased with age in the diabetic mice but not in the control mice (Figure 3A,D–F). Moreover, RAGE was co-localized with the macrophage marker F4/80 in the aged KK-Ay/TaJcl mice skin (Figure 3C,D). These findings indicated that skin dryness was affected by aging in diabetes mice.

### 3.4. Effect of Aging on PGE2, TNF-α, IL-1β, and COX-2 Skin Levels in KK-Ay/TaJcl Mice

**Figure 4:**
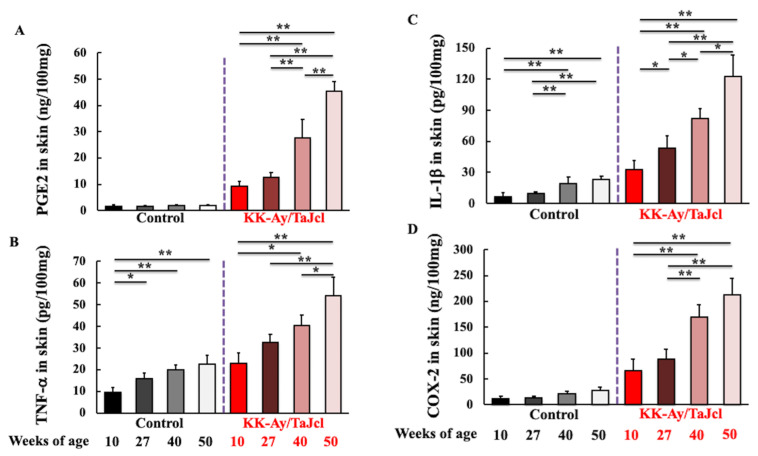
Effect of aging on the skin levels of PGE2 (**A**), TNF-α (**B**), IL-1β (**C**), and COX-2 (**D**) in KK-Ay/TaJcl mice evaluated using ELISA. Values are expressed as the mean ± SD of five animals. N = 5. ** *p* < 0.01; * *p* < 0.05. Control: C57BL/6j mice.

To evaluate whether inflammatory response was related to the exacerbation of skin dryness caused by aging, the skin levels of PGE2, TNF-α, IL-1β, and COX-2, which are secreted by macrophages, were measured. The levels of PGE2 (Figure 4A), TNF-α (Figure 4B), IL-1β (Figure 4C), and COX-2 (Figure 4D) were higher in KK-Ay/TaJcl mice than in control mice. Furthermore, PGF2, TNF-α, IL-1β, and COX-2 levels considerably increased with age in KK-Ay/TaJcl mice. Conversely, in control mice, these hardly increased with age.

### 3.5. Effect of Aging on MCP-1 and CCR2 Skin Expression in KK-Ay/TaJcl Mice

**Figure 5:**
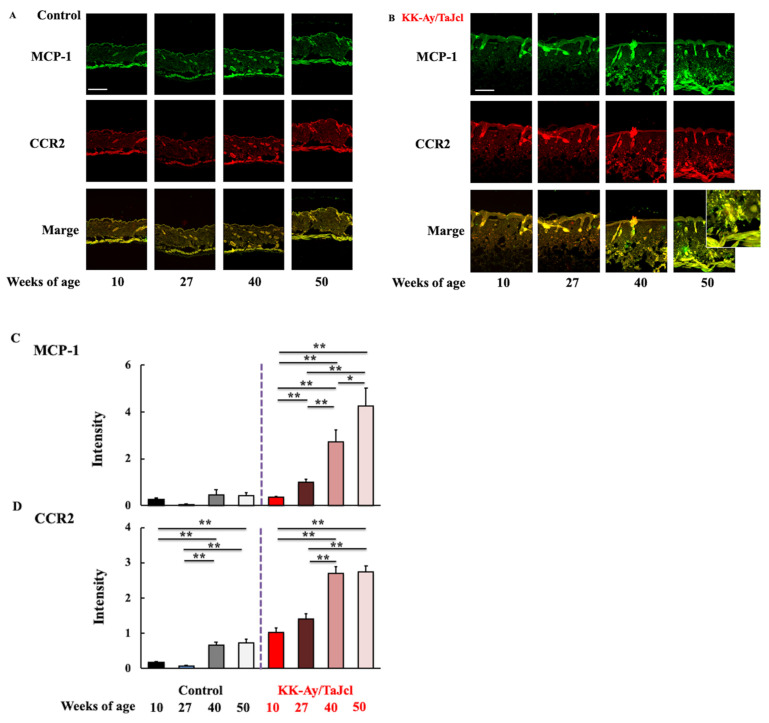
Effect of aging on the expression of MCP-1 (**A**,**C**) and CCR2 (**A**,**D**) and the co-localization of MCP-1 and CCR2 (**A**) in the dorsal skin of KK-Ay/TaJcl mice. In (**B**), a magnified view is inserted to confirm the co-localization of MCP-1 and CCR2. Scale bar = 100 μm. Values are expressed as the mean ± SD of five animals. N = 5. ** *p* < 0.01; * *p* < 0.05. Control: C57BL/6j mice.

Finally, we evaluated the expression of macrophage migration factors MCP-1 and CCR2 in the skin of control and diabetic mice. MCP-1 expression was higher and increased with age in KK-Ay/TaJcl mice, reaching the highest level at 50 weeks of age (Figure 5A–C). Similarly, CCR2 expression was higher and increased with age in KK-Ay/TaJcl mice; however, there was no change between 40 and 50 weeks of age (Figure 5A,B,D). However, in control mice, the expression of all migration factors was lower than that in KK-Ay/TaJcl mice, suggesting that macrophages actively migrated to skin in diabetic mice. Furthermore, CCR2 was co-localized with MCP-1 in aged KK-Ay/TaJcl mice skin (Figure 5A,B).

## 4. Discussion

Herein, we showed that skin dryness exacerbates with age in diabetic mice. Furthermore, we showed that AGE levels and the number of macrophages increase in the skin of aged diabetic mice and that RAGE expression also increases on the surface of these macrophages. Moreover, the skin levels of PGE2 and TNF-α increased, whereas collagen expression decreased in diabetic mice during aging. Finally, we showed an overexpression of macrophage migration factors MCP-1 and CCR2 in the skin of aged diabetic mice.

RAGE, the major receptor for AGEs, is a single-pass transmembrane protein belonging to the immunoglobulin superfamily and is known to mediate inflammatory responses by activating various intracellular signaling pathways [17,18]. As AGEs that bind to RAGE are present on retinal vascular endothelial cells and promote angiogenesis, they are considered as onset factors of proliferative diabetic retinopathy [17,19,20]. RAGE is expressed not only in blood-vessel-constituting cells but also in other cells, such as macrophages [21]. Herein, we showed that, in aged diabetic mice, the number of macrophages in the skin and RAGE expression on the surface of these macrophages is increased. AGEs bind to RAGE present on macrophages to activate these cells, which, in turn, produce PGE2, TNF-α, and IL-1β [7]. The production of PGE2 was markedly increased in the skin of aged diabetic mice. It might be possible that increased PGE2 production exacerbates skin damage by inducing inflammatory cell infiltration rather than enhancing vascular permeability in the skin. Furthermore, IL-1β released by macrophages induces COX-2 expression, resulting in the increased production of PGE2 [22]. Herein, the IL-1β and COX-2 skin levels were considerably increased in aged diabetic mice [22]. This suggests that a negative cycle of AGE/macrophage/RAGE/IL-1b/COX-2/PGE2 is induced in the skin of aged diabetic mice.

Previously, we reported that the secretion of inflammatory cytokines, especially TNF-α from the liver and kidney cells of diabetic mice, is increased during aging [23]. We speculate that PGE2 induces TNF-α leakage from the blood vessels of the skin, which further deteriorates skin dryness. In patients with diabetes, the accumulation of AGEs in the liver and kidneys is increased [24], indicating the possibility that AGEs are involved in the increased production of TNF-α in these organs.

We observed that the number of macrophages in the skin of diabetic mice increased during aging. Moreover, the expressions of MCP-1 and CCR2, both of which are involved in regulating macrophage migration [25], also increased in aged diabetic mice, indicating the active migration of macrophages to the skin. MCP-1 and CCR2 are secreted by adipocytes and other cells during obesity, which increases the infiltration of M1-polarized macrophages and stimulates the production of inflammatory cytokines [26]. We speculate that the increased expression of MCP-1 and CCR2 during aging might enhance the migration and accumulation of M1 macrophages, and increase the secretion of inflammatory cytokines. Thus, in aged diabetic mice, increased numbers of AGEs and macrophages and increased RAGE expression enhance the secretion of TNF-α, IL-1β, and PGE2 from macrophages. Thus, these molecules interact with each other to exacerbate skin dryness.

TNF-α, which plays an important role in regulating skin dryness, acts on insulin receptors present on the inner walls of blood vessels and inhibits their activity, resulting in a state of insulin resistance [27]. This inhibits sugar uptake by cells, resulting in a further increase in blood sugar levels and the aggravation of diabetes. Thus, AGE accumulation not only exacerbates dry skin but also exacerbates diabetes, leading to a negative spiral.

Research into the role of RAGE in skin diseases remains fairly limited. However, the involvement of RAGE and RAGE-related pathways in chronic inflammatory diseases and infectious diseases has been suggested [28]. These data warrant further research, may have clinical implications, and may help to better understand the pathogenesis of the disease. It would be greatly appreciated if this research is included in this series of studies despite its weaknesses.

The results of this study indicate that skin dryness is induced in aged diabetic mice and that AGE/macrophage/RAGE/PGE2-TNF-α-IL-1β pathways might be involved in the mechanism. However, further investigation is necessary to demonstrate this. Furthermore, this research cannot be directly applied to humans. Therefore, in future research, we are planning to conduct tests using a three-dimensional model of human skin, which mimic human responses, and perform age-related clinical trials using actual diabetes patients.

## 5. Conclusions

In KK-Ay/Tajcl mice, the dryness of the skin becomes worse with age. The AGE/macrophage/RAGE/PGE2-TNF-α-IL-1β pathways were shown to have some involvement in dry skin caused by type 2 diabetes. In addition, IL-1β increases the expression of PGE2, and TNF-α leads to insulin resistance and the accumulation of AGEs, exacerbating diabetes. Furthermore, damage to the liver and kidneys and the increased secretion of TNF-α from adipocytes aggravate not only dry skin but also diabetes. Therefore, when diabetes develops, it falls into a negative spiral, so it is thought to be most necessary to improve lifestyle habits and pay attention to diabetes.

## Data Availability

Not applicable.
